# Integrating music therapy and video games in cognitive interventions: innovative applications of closed-loop EEG

**DOI:** 10.3389/fnagi.2024.1498821

**Published:** 2024-12-18

**Authors:** Ying Wang, Kexin Zhang, Hao Yu, Xianglong Wan, Tiange Liu, Danyang Li, Dingna Duan, Xueguang Xie, Dong Wen

**Affiliations:** ^1^School of Information Science and Engineering, Yanshan University, Qinhuangdao, China; ^2^Department of Sports, University of Science and Technology Beijing, Beijing, China; ^3^School of Intelligence Science and Technology, University of Science and Technology Beijing, Beijing, China; ^4^Key Laboratory of Perception and Control of Intelligent Bionic Unmanned Systems, Ministry of Education, Institute of Artificial Intelligence, University of Science and Technology Beijing, Beijing, China

**Keywords:** music therapy, video games, cognitive function, EEG, combination

## 1 Introduction

Cognitive function refers to the mental abilities involved in acquiring, manipulating, and applying information, including but not limited to attention, memory, reasoning, language, spatial and executive functions (Ni et al., 2022). These cognitive abilities can deteriorate with age or due to certain medical conditions, leading to symptoms like memory loss, slowed thinking, and concentration difficulties (Unbehaun et al., 2021). Cognitive impairment often serves as an early indicator of diseases like Alzheimer's and Parkinson's disease (PD) (Litvan et al., 2011).

Therapeutic interventions like video games (VGs) and music therapy (MT) have demonstrated effectiveness in preventing or mitigating cognitive decline and enhancing cognitive function. VGs, known for their engaging and action-oriented nature, can foster improvements in cognitive skills through social and participatory elements (Wiley et al., 2021). In contrast, MT utilizes music to stimulate neuroplasticity, thereby enhancing cognitive function (Fang et al., 2017). The combination of MT and VGs provides a more robust therapeutic intervention than either approach alone, yielding a synergistic effect with substantial therapeutic potential (Martin-Moratinos et al., 2023). Music video games (MVGs) have also shown promise in preventing or mitigating cognitive decline and enhancing cognitive function.

In recent years, cognitive interventions based on electroencephalography (EEG) have gained considerable attention in psychology and neuroscience. These interventions utilize EEG to monitor brain activity, assess the effects of cognitive training (Rajakumar and Mohan, 2024), and guide the optimization of therapeutic strategies (Taya et al., 2015). Traditional open-loop EEG systems primarily record and analyze brain activity but lack a time feedback mechanism (Jin et al., 2024). In contrast, closed-loop EEG can monitor brain states in real time (Zhang et al., 2023), allowing for timely adjustments to intervention strategies and providing a more personalized and practical cognitive training experience (Dangi et al., 2013). As a result, closed-loop EEG-based music video game therapy (MVGT) shows excellent clinical potential in cognitive interventions.

This paper summarizes and analyzes cognitive intervention methods that combine MT and VGs, including the application of EEG in assessing intervention effectiveness. We explore EEG analysis techniques and present our perspective on the developmental trends of closed-loop EEG-based MVGT in cognitive interventions, aiming to offer valuable insights for future research.

## 2 Cognitive intervention combining music therapy and video games

### 2.1 Music therapy

MT stimulates various areas of the brain and promotes neural plasticity (Stegemoller, 2014). Active MT involves participants enhancing neural connections through learning instruments or engaging in music activities (Bégel et al., 2017). In contrast, passive MT activates sensory, motor, cognitive, language, and emotional regions in both hemispheres of the brain while participants listen to music performed by others (Moreira et al., 2023).

MT is an effective cognitive intervention that not only improves cognitive function (Xue et al., 2023) but also significantly reduces anxiety and depression (Lyu et al., 2018), enhancing overall quality of life. Factors such as music, rhythm, and task difficulty influence its effects (Schwartz et al., 2017). MT has been shown to benefits various patient populations, including those with dementia (Moreno-Morales et al., 2020), attention deficit hyperactivity disorder (Martin-Moratinos et al., 2023), and PD (Barnish and Barran, 2020), effectively enhancing their cognitive abilities.

However, despite its advantages, MT typically requires guidance from a professional music therapist (Li et al., 2015). Participants often need a therapist to implement effective MT independently (Li et al., 2019), which limits their ability to apply MT into daily life.

### 2.2 Video game therapy

A comprehensive 68-year study has shown that moderate participation in VGs between the ages of 11 and 79 can enhance cognitive abilities (Altschul and Deary, 2020). The research found that video game training significantly improves cognitive function in older adults, particularly in visual-spatial working memory and episodic memory. Additionally, video game therapy has shown positive outcomes in patients, markedly enhancing overall cognitive function in individuals with schizophrenia (Shi et al., 2023) and improving executive function and information processing speed in those with mild cognitive impairment (Menascu et al., 2021).

However, during cognitive interventions involving gaming, while players may experience improvements in specific cognitive skills, they may also experience negative emotional outcomes (Miller and Mandryk, 2016). Prolonged gaming can result in fatigue, anxiety, or frustration (Kuperczko et al., 2022), especially when confronting challenging tasks. If these emotional issues are not addressed, they may adversely affect the players' overall experience and mental health.

### 2.3 Music video game therapy

Integrating music with VGs through MVGT presents a promising avenue for cognitive intervention. Music is closely linked to emotions, motivation, and reward within the gaming context (Loizou et al., 2014). By designing music-based VGs, players can enjoy an engaging experience while benefiting from the therapeutic qualities of music (Ticker, 2017). Research indicates that MVGs, which combines music as a motivating factor with gameplay mechanics, can effectively improve memory and spatial awareness in patients with cognitive impairments (Vargas et al., 2020), making them suitable for long-term training.

Moreover, MVGs also facilitate coordination between auditory and motor skills. For instance, rhythm games can be utilized in rehabilitation training for patients with PD (Dalla Bella, 2022), helping to improve their motor abilities. Music also aids participants in concentrating better and focusing on specific game tasks. In a music attention training game, users must follow rhythm and speed, following the music to perform and improvise (Chalkiadakis, 2022). Research indicates that this type of game significantly enhances participants' selective attention, underscoring the considerable potential of MVGs in cognitive intervention.

Overall, by combining the benefits of music therapy and video games, MVGs represent a promising approach to enhancing cognitive abilities in participants. Despite their significant potential, MVGT faces important limitations that must be addressed. For instance, personalized treatment plans and standardized procedures for MVGs still need to be fully developed. Additionally, variations in players' musical and gaming preferences and learning styles may affect the effectiveness of interventions (Liu et al., 2016). Therefore, it is essential to develop innovative assessment and guidance strategies to optimize the implementation process of MVGs.

## 3 The integration of electroencephalography and music video game therapy

Cognitive function is closely linked to neural connections between different brain regions (Park and Friston, 2013). EEG provides real-time insights into brain activity and is widely recognized as an essential indicator for assessing changes in cognitive abilities (van der Hiele et al., 2007). By analyzing EEG signals, researchers can detect physiological changes in patients' brains (Ribas et al., 2013), enbaling a more objective evaluation of treatment effects. The combination of EEG with MVGs offers a fresh perspective for cognitive assessment and intervention guidance. Two main approaches for integrating EEG with MVGs are open-loop and closed-loop EEG systems, each presenting unique opportunities for cognitive intervention.

Research using open-loop EEG with MVGs demonstrates significant improvements in cognitive function. For instance, one study involved participants engaged in MVGs, while researchers analyzed their EEG data to compare power spectral density and individual alpha frequency to assess changes in attention levels (GomezRomero-Borquez et al., 2023). This study examined the alpha asymmetry between the left and right hemispheres of the brain. The results showed that MVGs significantly enhanced participants' attention. Additionally, another study revealed that compared to casual social games, music rhythm games elicited higher theta/alpha EEG values (Ribas et al., 2013). This research also revealed enhanced connectivity in the alpha and beta1 bands between the bilateral temporal lobes. In contrast, connectivity between the frontal and occipital lobes decreased, indicating that participants exhibited higher attention levels and greater focus on auditory attention during music rhythm games.

While these findings highlight the potential of MVGs for cognitive intervention, practical applications require dynamic adjustments to the game training content based on participants' cognitive states (Mortazavi et al., 2024) to maximize the game's effectiveness and achieve optimal cognitive intervention outcomes. However, open-loop EEG cannot provide real-time feedback on cognitive states (Farkhondeh Tale Navi et al., 2022), limiting the ability to dynamically assess and adjust player performance during gameplay, which hampers responsiveness to changes in cognitive needs (Ehrlich et al., 2019). Consequently, future research should explore more effective real-time feedback mechanisms to enhance the flexibility and effectiveness of cognitive interventions.

In contrast, EEG-based closed-loop music video game therapy leverages real-time feedback, offering multiple advantages in clinical applications. The closed-loop system captures participants' EEG signals in real-time, monitoring changes in attention (Li et al., 2011), emotional state (Ehrlich et al., 2019), and cognitive load (Koenig et al., 2011) and dynamically adjusting the game's difficulty or the music's tempo based on these signals (Schlette, 2023). This real-time feedback mechanism ensures flexibility and adaptability in the intervention. Moreover, the collected EEG signals can be utilized for Neurofeedback Training, enhancing the overall effectiveness of cognitive training (Jirayucharoensak et al., 2019). Research indicates that this training yields significantly better outcomes than interventions without Neurofeedback Training. Additionally, the closed-loop system can design personalized treatment plans based on the unique EEG patterns of each participant (Carè et al., 2024), thereby improving the specificity and effectiveness of the intervention. Therefore, EEG-based closed-loop music video game cognitive interventions provide participants with more personalized and effective rehabilitation strategies.

In summary, the closed-loop system, through real-time monitoring and feedback, can more accurately address the specific needs of cognitive interventions, thereby enhancing the effectiveness of music video game therapy. However, EEG-based music video games remain a relatively new research area in cognitive interventions, with limited research and applications currently available.

## 4 Current challenges and future research prospects

Research indicates that EEG plays a crucial role in enhancing the effectiveness of cognitive interventions (Wan et al., 2024), particularly as closed-loop EEG can provide personalized treatment plans for each participant. However, several challenges must be addressed to fully realize this adaptive and personalized treatment level.

### 4.1 The importance of cross-subject and cross-task EEG analysis

EEG signals exhibit high variability and non-stationarity across subjects (Jimenez-Guarneros and Gomez-Gil, 2020), which can lead to mismatched data distributions among samples. Current research primarily focuses on single tasks, which limits the generalization capability of EEG analysis models to other tasks within the same cognitive domain (Zhong et al., 2024). Therefore, cross-subject and cross-task EEG signal analysis is essential for effectively assessing the impact of cognitive interventions. The cross-subject analysis utilizes data from other individuals to calibrate a new subject (Wu et al., 2022). In contrast, cross-task analysis employs labeled data from similar or related tasks to calibrate new tasks.

To evaluate the effects of EEG-based cognitive interventions more effectively, it is crucial to establish a general EEG analysis model applicable across different subjects and tasks within the same cognitive domain (Zhou et al., 2022). By integrating closed-loop EEG technology, EEG signals can be analyzed more accurately across different subjects and tasks (Walter, 2015). Recording the EEG patterns of subjects during specific cognitive tasks and comparing the signals of different subjects performing the same task can help identify critical factors influencing cognitive performance (Jimenez-Guarneros and Gomez-Gil, 2020). Additionally, analyzing EEG signals from different cognitive tasks helps reveal the dynamic changes in how the brain processes various types of information (Wen et al., 2023). This approach enhances data collection flexibility and provides essential support for personalized cognitive intervention programs.

### 4.2 Individualization of intervention programs

The individualized intervention plan is designed to adjust the selection of music and VGs based on each patient's cognitive needs and abilities (Cicerone et al., 2019). Different music and game content types may have varying effects on each participant (Choi et al., 2020), making a tailored approach significantly more effective for cognitive intervention (Buzzi et al., 2019). In this process, closed-loop EEG technology plays a crucial role by allowing real-time monitoring and analysis of each participant's brain signals, providing precise feedback for personalized interventions (Carè et al., 2024). This method enhances patient engagement and satisfaction and facilitates the smooth treatment progress. By utilizing closed-loop EEG, we can more effectively identify individual differences, optimize intervention plans, and improve the effectiveness and sustainability of the interventions.

### 4.3 Effectiveness of integrated cognitive intervention therapies

Future research should focus on the effectiveness of integrated intervention therapies (Jung et al., 2020) to explore their impact on cognitive function. Studies have shown that combining music video games with cognitive training can significantly enhance short-term memory, attention, and inhibitory control in older adults (Lin et al., 2020). Furthermore, integrating music games with exercise therapy has been found to promote social interaction, engagement, and memory recall among dementia patients with dementia (Unbehaun et al., 2021). Therefore, it is recommended that future studies conduct further investigations into integrated cognitive interventions to assess their potential to enhance cognitive function systematically. Comprehensive cognitive intervention therapy will help us understand the impact of different cognitive intervention therapies on cognitive training to optimize the intervention program and promote improving cognitive health.

## 5 Conclusion

In conclusion, closed-loop EEG-based MVGT presents promising opportunities for cognitive intervention. We recommend collecting participants' EEG data during the interventions to facilitate ongoing adjustments and monitor the progress of treatment and rehabilitation. Future research should focus on the following key themes: (1) cross-subject and cross-task EEG signal analysis; (2) personalized intervention therapies; (3) integrated cognitive intervention therapies; (4) obtaining sufficient experimental data; (5) exploring the relationship between EEG signal features and task performance. From our perspective, in-depth research in this field will contribute to more effective cognitive intervention methods.
